# Commentary: Euthyroid Sick Syndrome in Patients With COVID-19

**DOI:** 10.3389/fendo.2021.633097

**Published:** 2021-02-10

**Authors:** Annunziatina Laurino, Manuela Gencarelli, Lisa Buci, Laura Raimondi

**Affiliations:** ^1^ Department of Neuroscience, Psychology, Drug Sciences, and Child Health (NEUROFARBA), University of Florence, Florence, Italy; ^2^ Endocrinology Unit, Careggi Hospital, Florence, Italy

**Keywords:** thyromimetics, thyroid diseases, thyroid hormone, COVID-19 pandemic, anosmia, sarcopenia

Recent data from case reports and small clinical studies indicates thyroid disorders characterized by reduction of T3 levels are found associated with adverse events and all causes of mortality in Covid-19 patients (1–7). In this scenario the paper from Zuo et al. (4) consistently describes the presence of the euthyroid sick syndrome (EES) correlating with Covid-19 disease severity but not with non-invasive or invasive ventilation. None of these reports clarify the clinical meaning of these endocrine disturbances, and their pathogenesis remains elusive. In this respect, hypotheses include they represent a stress-evoked response or are generated by a direct attack of the virus to the gland. This latter hypothesis is sustained by findings demonstrating gland cell types can be target of viruses from bat origin. Consistently, it has been reported that i) human thyrocytes express the mRNA for ACE2 (8), the host receptor of the virus Spike protein, ii) viral particles were detected in the follicular epithelium of patients who died of SARS and also of Covid-19 presenting subacute thyroiditis (9) iii) Covid-19 patients may present, where measured, high levels of pro-calcitonin (PCT). Overall, these intriguing data would need to be confirmed by larger clinical studies to establish conclusively the causal relationship, if any, among thyroid diseases and virus infection, and, more importantly, their clinical relevance as possible prognostic indicators of mortality and of severe cardiovascular events (10). However, in the light of the present data, we like to share some considerations on the possibility that reduced serum T3 levels, as observed in EES, because they are not representative of its tissue-specific levels, coincide with accumulation of T3 metabolites, *i.e.* thyromimetics. The accumulation of such compounds in the olfactory epithelium and skeletal muscle could offer a pathogenic hypothesis for the onset of anosmia and sarcopenia, two fingerprint manifestations of Covid-19 infection.

In extra thyroid tissues, T3 levels are controlled by type 1, 2, and 3 deiodinases (DIO1, DIO2, and DIO3 respectively), enzymes removing iodide ions with respect to their position on the aromatic rings and that have a high specific tissue expression. In particular, DIO2 and DIO3 are the main isoforms expressed in neurons and skeletal muscle, and they are rapidly modulated at inflammatory conditions, including ESS (4) and, in general, infective agent attacks (11, 12). At conditions of reduced DIO2 activity, T4 metabolism is mainly driven towards revT3, the alternative metabolite with a very low intrinsic activity at thyroid hormone receptors and considered among the source of other iodinated metabolites belonging to three different chemical classes including thyronamines and thyroacetic acids. These metabolites are indicated as endogenous thyromimetics accumulating in thyroid hormone target tissues and sharing the same but not all the biological effects of T3 (13). Among these compounds, the 3-iodothyronamine (T1AM) is the more characterized in term of pharmacokinetic and pharmacodynamics features (13). Pharmacologically administered T1AM elicited neurological and metabolic effects with high potency. The mechanism of several of these effects remains to be clarified, and possible targets have been described including the trace amine associated receptors (TAAR1-5), while its affinity at thyroid hormone receptors is negligible. TAARs are evolutionarily conserved in vertebrates including humans, exerting an indispensable role in olfaction (14). T1AM, and possibly other thyronamines by the means of their nature as “amines in trace”, is a high affinity ligand for TAAR1 and is described as an inverse agonist at the TAAR5 (15). The olfactory epithelium expresses several TAAR isoforms including TAAR5 but also ACE2 and TMPRSS2, the host virus receptors. According to its pharmacodynamics, T1AM is expected to reduce the basal activity of TAAR5. Down-regulation of TAARs (and anosmia) is reported as a secondary event to immune innate signaling activation induced by virus infection (16). It is then possible to postulate accumulation of T1AM, as result of an increase of revT3 concentration, might have a role in increasing the threshold of olfactory receptor activation thus participating to Covid-19 induced anosmia. The fast recovery from this adverse event experienced by some Covid-19 patients would be consistent with the short half-life of T1AM. In those where anosmia persists even after infection resolution, desensitization effects could add to more complex damages at neurological circuits.

Sarcopenia associated with Covid-19 is likely secondary to the cytokine storm and of a long bed rest even if a direct attack of the virus to the skeletal myocytes cannot be excluded since satellite cells and adult myofibers express ACE2 (17). Sarcopenia has a negative impact on patient recovery (18), and it represents a negative prognostic factor for cardiovascular complications (19). Since T3 strongly controls skeletal muscle regenerative capacity and metabolic functions (20), reduced levels of T3 together with vascular inflammation and cachexia, might trigger skeletal muscle catabolism and an incorrect myogenic program of satellite cells. In addition, a role for calcitonin in controlling satellite cell quiescence and their escape from fiber niche, a condition exposing satellite cells to aging and a fibrotic fate, has been described recently (21). In the skeletal muscle, Ju et al. (2017) reported T1AM activated catabolic pathways involved in sarcopenia including AMPK activation (22).

Overall, anosmia and sarcopenia have an inflammatory ground which includes a crucial balance between DIO2/DIO3, thus controlling T3 local production (23, 24). Accumulation of revT3 and of T1AM may act in concert as pathogenic events in these Covid-19 clinical manifestations.

The role of thyromimetics in Covid-19 related anosmia and sarcopenia is a hypothesis which needs to be confirmed by clinical data. In this respect we launched the opportunity to include the evaluation of thyromimetics among the biomarkers of the thyroid function. Covid-19 pandemic might offer an extraordinary opportunity for investigating the role of the thyroid and to assign a physiopathological role to endogenous thyromimetics.

## Author Contributions

AL and LR wrote the paper. MG collected the references. LB supervised the paper. All authors contributed to the article and approved the submitted version.

## Funding

The paper was supported by Ente Cassa di Risparmio di Firenze and by a local grant from the University of Florence.

## Conflict of Interest

The authors declare that the research was conducted in the absence of any commercial or financial relationships that could be construed as a potential conflict of interest.

## References

[1] Caron P. Thyroid disorders and SARS-CoV-2 infection: From pathophysiological mechanism to patient management. Ann Endocrinol (Paris) (2020) 81:507–10. 10.1016/j.ando.2020.09.001 PMC7498405 32950466

[2] Ruggeri RM Campennì A Siracusa M Frazzetto G Gullo D. Subacute thyroiditis in a patient infected with SARS-COV-2: an endocrine complication linked to the COVID-19 pandemic. Hormones (Athens) (2020). 10.1007/s42000-020-00230-w PMC7365600 32676935

[3] Brancatella A Ricci D Cappellani D Viola N Sgrò D Santini F. Is Subacute Thyroiditis an Underestimated Manifestation of SARS-CoV-2 Infection? Insights From a Case Series. J Clin Endocrinol Metab (2020). 10.1210/clinem/dgaa537 PMC7454668 32780854

[4] Zou R Wu C Zhang S Wang G Zhang Q Yu B. Euthyroid Sick Syndrome in Patients With COVID-19. Front Endocrinol (Lausanne) (2020). 10.3389/fendo.2020.566439 PMC7575767 33117282

[5] Ippolito S Dentali F Tanda ML. SARS-CoV-2: a potential trigger for subacute thyroiditis? Insights from a case report. J Endocrinol Invest (2020). 10.1007/s40618-020-01312-7 PMC7266411 32488726

[6] Muller I Cannavaro D Dazzi D Covelli D Mantovani G Muscatello A. SARS-CoV-2-related atypical thyroiditis. Lancet Diabetes Endocrinol (2020). 10.1016/S2213-8587(20)30266-7 PMC7392564 32738929

[7] Gao W Guo W Guo Y Shi M Dong G Wang G. Thyroid hormone concentrations in severely or critically ill patients with COVID-19. J Endocrinol Invest (2020). 10.1007/s40618-020-01460-w PMC7605732 33140379

[8] Rotondi M Coperchini F Ricci G Denegri M Croce L Ngnitejeu ST. Detection of SARS-COV-2 receptor ACE-2 mRNA in thyroid cells: a clue for COVID-19-related subacute thyroiditis. J Endocrinol Invest (2020). 10.1007/s40618-020-01436-w PMC7538193 33025553

[9] Desaillud R Hober D. Virus and thyroiditis: an update. Virol J (2009). 10.1186/1743-422X-6-5 PMC2654877 19138419

[10] Barrera FJ Shekhar S Wurth R Moreno-Pena PJ Ponce OJ Hajdenberg M. Prevalence of Diabetes and Hypertension and Their Associated Risks for Poor Outcomes in Covid-19 Patients. J Endocr Soc (2020). 10.1210/jendso/bvaa102 PMC7454711 32885126

[11] Xu M Iwasaki T Shimokawa N Sajdel-Sulkowska EM Koibuchi N. The effect of low dose lipopolysaccharide on thyroid hormone-regulated actin cytoskeleton modulation and type 2 iodothyronine deiodinase activity in astrocytes. Endocr J (2013). 10.1507/endocrj.ej13-0294 23965412

[12] Wajner SM Maia AL. New Insights toward the Acute Non-Thyroidal Illness Syndrome. Front Endocrinol (2012). 10.3389/fendo.2012.00008 PMC3356062 22654851

[13] Zucchi R. Thyroid Hormone Analogues: An Update. Thyroid (2020). 10.1089/thy.2020.0071 PMC7415871 32098589

[14] Dewan A Cichy A Zhang J Miguel K Feinstein P Rinberg D. Single olfactory receptors set odor detection thresholds. Nat Commun (2018). 10.1038/s41467-018-05129-0 PMC6056506 30038239

[15] Dinter J Mühlhaus J Wienchol CL Yi CX Nürnberg D Morin S. Inverse agonistic action of 3-iodothyronamine at the human trace amine-associated receptor 5. PloS One (2015) 10:e0117774. 10.1371/journal.pone.0117774 25706283 PMC4382497

[16] Rodriguez S Cao L Rickenbacher GT Benz EG Magdamo C Ramirez Gomez LA. Innate immune signaling in the olfactory epithelium reduces odorant receptor levels: modeling transient smell loss in COVID-19 patients. (2020) 2020.06.14.20131128. 10.1101/2020.06.14.20131128

[17] Laurino A Spinelli V Gencarelli M Balducci V Dini L Diolaiuti L. Angiotensin-II Drives Human Satellite Cells Toward Hypertrophy and Myofibroblast Trans-Differentiation by Two Independent Pathways. Int J Mol Sci (2019) 20:4912. 10.3390/ijms20194912 PMC6801484 31623362

[18] Morley JE Kalantar-Zadeh K Anker SD. COVID-19: a major cause of cachexia and sarcopenia? J Cachexia Sarcopenia Muscle (2020). 10.1002/jcsm.12589 PMC7300782 32519505

[19] Xia MF Chen LY Wu L Ma H Li XM Li Q. Sarcopenia, sarcopenic overweight/obesity and risk of cardiovascular disease and cardiac arrhythmia: A cross-sectional study. Clin Nutr (2020). 10.1016/j.clnu.2020.06.003 32593523

[20] Bloise FF Oliveira TS Cordeiro A Ortiga-Carvalho TM. Thyroid Hormones Play Role in Sarcopenia and Myopathies. Front Physiol (2018). 10.3389/fphys.2018.00560 PMC5992417 29910736

[21] Yamaguchi M Watanabe Y Ohtani T Uezumi A Mikami N Nakamura M. Calcitonin Receptor Signaling Inhibits Muscle Stem Cells from Escaping the Quiescent State and the Niche. Cell Rep (2015). 10.1016/j.celrep.2015.08.083 26440893

[22] Ju H Kim T Chung CM Park J Nikawa T Park K. Metabolic Suppression by 3-Iodothyronamine Induced Muscle Cell Atrophy via Activation of FoxO-Proteasome Signaling and Downregulation of Akt1-S6K Signaling. Biol Pharm Bull (2017). 10.1248/bpb.b16-00653 28163294

[23] Luongo C Dentice M Salvatore D. Deiodinases and their intricate role in thyroid hormone homeostasis. Nat Rev Endocrinol (2019). 10.1038/s41574-019-0218-2 31160732

[24] Mancini A Di Segni C Raimondo S Olivieri G Silvestrini A Meucci E. Thyroid Hormones, Oxidative Stress, and Inflammation. Mediators Inflamm (2016) 2016:6757154. 10.1155/2016/6757154 27051079 PMC4802023

